# Metallic Casts

**Published:** 1858-08

**Authors:** C. P. Baird

**Affiliations:** Winchester, Tenn.


Winchester, Tenn., May 19, 1858.

My Dear Friend Toland:—Your "Dental Reporter" has just reached us, and we are highly pleased with it. I have lately made an improvement in my manner of taking a metallic cast from the plaster, which may not be new to many of your readers; but if it should be of any benefit to any of them they are welcome to the trouble of my few lines. I have taken nineteen smooth and perfect casts and not failed once. And I can do the whole work whilst any one will be making the preparation for it in sand, and it is far more certain than the dipping process. Prepare you a small sheet-iron box, round or square, as you like—say four inches in diameter, three and a half or four high, and smaller at bottom than top—then take your plaster cast, and after giving it the shape you want, cut it away on the bottom until it is quite thin; this will facilitate the drying and make it take less lead. Now dry it perfectly, put on your lead, and when melted set your cast on it and bring it to as near the same temperature with the lead as you can. Now set your box near by and pour in your metal until the bottom is covered—say one inch deep—set your plaster cast on its surface with teeth up, and press it down into the melted metal until it almost runs into the palate, and when the metal cools sprinkle a little dry plaster over it; this will keep it from uniting—blow off the plaster that may have fallen on the cast. Now take the lead, cool as it will pour, and pour it on the cast slowly until it is burned three-quarters or an inch deep, take them out of your box and you will have a complete cast. Take the male cast the usual way. I use lead for female, and block tin for male casts. This is as good as I care to have. I have no trouble at all in getting a perfect cast when I want it.

Yours, C. P. Baird.

